# Poststroke Neuropsychiatric Symptoms: Relationships with IL-17 and Oxidative Stress

**DOI:** 10.1155/2014/245210

**Published:** 2014-06-18

**Authors:** W. Swardfager, N. Herrmann, A. C. Andreazza, R. H. Swartz, M. M. Khan, S. E. Black, K. L. Lanctôt

**Affiliations:** ^1^Neuropsychopharmacology Research Group, Sunnybrook Research Institute, 2075 Bayview Avenue, Toronto, ON, Canada M4N 3M5; ^2^Department of Psychiatry, Sunnybrook Health Sciences Centre, 2075 Bayview Avenue, Toronto, ON, Canada M4N 3M5; ^3^Canadian Partnership for Stroke Recovery, Sunnybrook Health Sciences Centre, 2075 Bayview Avenue, Toronto, ON, Canada M4N 3M5; ^4^Division of Cognitive Neurology, Department of Medicine, Sunnybrook Health Sciences Centre, Toronto, ON, Canada M4N 3M5; ^5^L.C. Campbell Cognitive Neurology Research Unit, Sunnybrook Research Institute, Toronto, ON, Canada M4N 3M5; ^6^Department of Psychiatry, University of Toronto, 250 College Street, 8th Floor, Toronto, ON, Canada M5T 1R8

## Abstract

Stroke variably activates interleukin- (IL-) 17 expression, reduces regulatory T cells, and induces oxidative stress, which may support neurodegeneration. Ischemic stroke patients were screened for depressive symptoms (Center for Epidemiological Studies Depression (CES-D)) and cognitive status (Mini Mental State Examination). Proinflammatory cytokines (IL-17, IL-23, and interferon- [IFN-] *γ*), anti-inflammatory cytokine IL-10, and lipid hydroperoxide (LPH), a measure of oxidative stress, were assayed from fasting serum. Of 47 subjects (age 71.8 ± 14.4 years, 36% female), 19 had depressive symptoms (CES-D ≥ 16), which was associated with poorer cognitive status (*F*_1,46_ = 8.44, *P* = 0.006). IL-17 concentrations did not differ between subjects with and without depressive symptoms (*F*_1,46_ = 8.44, *P* = 0.572); however, IL-17 was associated with poorer cognitive status in subjects with depressive symptoms (*F*_1,46_ = 9.29, *P* = 0.004). In those subjects with depressive symptoms, IL-17 was associated with higher LPH (*ρ* = 0.518, *P* = 0.023) and lower IL-10 (*ρ* = −0.484, *P* = 0.036), but not in those without. In conclusion, poststroke depressive symptoms may be associated with cognitive vulnerability to IL-17 related pathways, involving an imbalance between proinflammatory and anti-inflammatory activity and increased oxidative stress.

## 1. Introduction

Stroke is a leading cause of disability, and common sequelae such as depression and cognitive impairment contribute significantly to disease burden among survivors. Depression after stroke has been associated with cognitive impairment, as assessed using the Mini Mental State Examination (MMSE) [1, 2]; however, biological mechanisms that may mediate this relationship remain elusive. Depression in medically healthy patients has been associated with increased concentrations of cytokines in peripheral blood [3], which may be relevant to depression after stroke [4]. Previous studies have identified relationships between MMSE scores and peripheral blood inflammatory markers, including C-reactive protein and kynurenine [5, 6], suggesting inflammation as a possible link between depression and cognitive impairment after stroke.

In animal models, the infiltration of T cells that express IL-17 exacerbates neurodegenerative damage in the delayed phase of postischemic injury [7]. In the peri-infarct cortex, apoptosis is the predominant mode of neuronal death, which is heavily influenced by inflammatory and anti-inflammatory cytokine signals released from infiltrating peripheral T lymphocytes and other cell types; however, only a few clinical studies have investigated IL-17 after stroke [8, 9]. In one study, IL-17 expression by peripheral mononuclear cells was associated with poorer neurological outcomes, although relationships with depression and cognitive status were not assessed [10]. IL-17 can induce blood brain barrier disruption through a mechanism that depends on the production of reactive oxygen species [11], suggesting that IL-17 could exacerbate neurodegeneration through oxidative damage to lipids, protein, and DNA. Recently, behavioral effects of IL-17 were demonstrated in an animal study, which reported that IL-17 expressing T cells exacerbated behavioral deficits during experimental induction of depression-like behaviors [12]. Based on those findings, it was hypothesized that serum IL-17 concentrations would be associated with depressive symptoms and cognitive impairment following acute ischemic stroke. This study explored relationships between IL-17, cognition, depression, and lipid peroxidation.

## 2. Materials and Methods

This cross-sectional observational study recruited consecutive English-speaking participants admitted to an acute care regional stroke centre with verified acute ischemic infarctions on CT or MR imaging. Patients with a medical history of prestroke dementia, hemorrhagic stroke, decreased consciousness, severe aphasia or dysarthria, significant acute medical or neurological illness other than stroke, and presence of a premorbid diagnosis of an axis I psychiatric disorder other than unipolar depression or chronic medical conditions known to have an inflammatory component were excluded. The protocol was approved by the Research Ethics Board at Sunnybrook Health Sciences Centre. All participants provided written informed consent prior to participation.

Depression was screened using the Center for Epidemiological Studies Depression Scale (CES-D) on which a score ≥16 is a reliable and sensitive indicator of poststroke depression [13]. Medical comorbidity has been found not to interfere with the accuracy of the CES-D to screen depressive episodes [14]. A trained researcher administered the CES-D scale (a self-report instrument assessing the presence and severity of symptoms over the past week) under the supervision of the study psychiatrist. Cognitive status was assessed using the MMSE, which has been validated in stroke [15], and administered by experienced personnel trained by the study psychiatrist. Stroke severity was assessed using the National Institutes of Health Stroke Scale (NIHSS) [16]. The CES-D, MMSE, and NIHSS were administered either on the same day as the blood draw or on the afternoon before. For patients with an available clinical CT scan, lesion location was recorded, stroke lesions were traced, and lesion volumes were recorded.

Within 24 hours of assessment, fasting blood was collected via venipuncture in BD SST Vacutainer (New Jersey, USA) tubes at 7:30 am ± 30 minutes. Serum was separated and stored at −80°C until analyzed. The proinflammatory Th17 cytokines IL-17 and IL-23 were assayed by standard enzyme linked immunosorbent assays according to manufacturers' instructions (Abcam, Toronto, ON, Canada). The Th1 effector cytokine interferon- [IFN-] *γ* and the anti-inflammatory cytokine IL-10 were measured using a multiplex suspension bead array immunoassay (Luminex Corporation, Austin, TX, USA). The intra-assay variabilities for the ELISA and Luminex kits were less than 15% (for the IL-17 assay, the coefficient of variability was 3.3%). All analyses were performed in a single batch to avoid variability between assays. Assay sensitivities were 0.2 pg/mL for IL-10, 1.0 pg/mL for IFN-*γ*, 20 pg/mL for IL-23, and 3 pg/mL for IL-17. Undetectable serum concentrations were imputed at their lower limits of detectability for subsequent analyses. As a stable measure of oxidative stress, lipid hydroperoxides (LPH) were assayed spectrophotometrically as described previously [17].

The Kolmogorov-Smirnov test was used to assess normality of distribution. Serum analyte concentrations and lesion volumes were log transformed to obtain normal distributions for use in analyses of covariance (ANCOVA). Patient characteristics were compared between those with and without depressive symptoms using* t*-tests for continuous measures and Chi-square tests for categorical variables. MMSE scores were compared to patient characteristics using* t*-tests for categorical variables or Pearson correlations for continuous measures. Variables related to IL-17 concentrations, depressive symptoms, or MMSE scores at trend level were controlled for systematically in models post hoc. Because differential associations between inflammatory and oxidative stress markers have been observed between depressed and nondepressed subjects [18, 19], relationships between serum markers in subgroups of patients with and without depressive symptoms were explored in Spearman correlations, due to smaller sample sizes.

Sample size was chosen based on effect sizes observed previously relating MMSE scores and serum inflammatory markers [5, 6]. Statistical analyses were performed using SPSS statistical software (version 20; SPSS Inc., Chicago, Illinois) or in R (http://www.R-project.org/).

## 3. Results

382 patients following ischemic stroke were screened for the study and 138 patients were carefully selected who met inclusion criteria and did not meet any criterion for exclusion. A total of 47 patients (aged 71.8 ± 14.4, 36% female) with mild to moderate stroke severity (NIHSS scores 4.9 ± 4.5) who agreed to participate and who had available serum samples were included in this analysis. Nineteen patients screened positive for depressive symptoms (CES-D ≥ 16), and subjects with and without depressive symptoms were similar in demographics and clinical characteristics (Table 1) although trends were noted for age, hypertension, dyslipidemia, and lesion location. Subjects with and without depressive symptoms did not differ in serum markers (Table 2).

The mean MMSE score was 27.1 ± 2.8. MMSE scores were associated with IL-17 concentrations in patients with depressive symptoms (*r* = −0.493, *P* = 0.032). MMSE scores were not associated with other patient characteristics (Table 1) or serum markers (Table 2).

Serum cytokine and LPH concentrations are presented in Table 2. No relationships were observed between the serum analytes and patient characteristics from Table 1, including ASA or NSAID use, NIHSS scores, and time since stroke (*P* > 0.05). Most patients included in the study had a large-artery atherosclerosis. No correlations were found between serum levels of biomarkers and etiologic origin of ischemic stroke. Lesion volume was correlated with IFN-*γ* (*ρ* = 0.363, *P* = 0.03) in this cohort, but not with any other serum analyte.

To test the hypothesis that IL-17 concentrations are associated with depressive symptoms, an ANCOVA model to assess differences in IL-17 concentrations between those with and without depressive symptoms, controlling for age and gender, was used. Serum IL-17 concentrations did not differ between patients with and without depressive symptoms (*F*_1,46_ = 0.342, *P* = 0.572).

**Figure 1:**
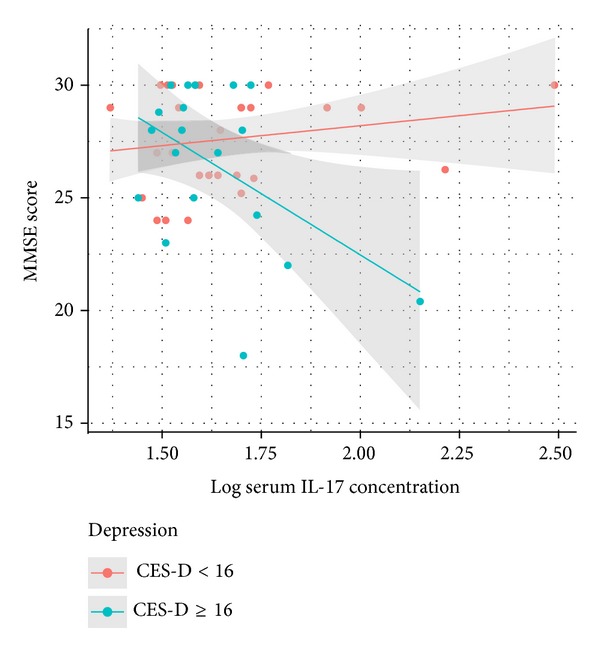
Differential association between peripheral IL-17 concentrations and cognitive status between patients with and without depressive symptoms. MMSE: Mini Mental State Examination, IL-17: interleukin-17, and CES-D: Center for Epidemiological Studies Depression Scale.

To test the hypothesis that IL-17 concentrations were associated with MMSE scores, an ANCOVA model predicting MMSE scores controlling for age, gender, and depression was used. Depression was associated with poorer MMSE scores (*F*_1,46_ = 8.44, *P* = 0.006) and there was a significant depression × IL-17 interaction (*F*_1,46_ = 9.29, *P* = 0.004) whereby IL-17 concentrations were associated with poorer cognitive status in patients with depressive symptoms (see Figure 1). The model explained 15.9% of the variance in MMSE scores. The interaction between depressive symptoms and IL-17 in predicting MMSE scores persisted in post hoc models controlling for hypertension, dyslipidemia, history of depression, antidepressant use, NSAID use, lesion location, NIHSS scores, and time between phlebotomy and assay.

In Spearman correlations, serum IL-17 concentrations were associated with higher LPH concentrations in patients with depressive symptoms (*ρ* = 0.518, *P* = 0.023), but not in those without (*ρ* = 0.107, *P* = 0.587). IL-17 was also associated with lower serum IL-10 concentrations in patients with depressive symptoms (*ρ* = −0.484, *P* = 0.036) but not in those without (*ρ* = −0.100, *P* = 0.611). Similar relationships were not observed with IL-23 or IFN-*γ*.

## 4. Discussion

In this study, poststroke depression was not associated with a bias towards peripheral production of IL-17. Relationships between poststroke depression and peripheral concentrations of other cytokines have been inconsistent, with increases noted in some [20–22] but not all previous studies [23–25]. However, in the present study poststroke depression was associated with cognitive impairment, replicating findings from previous studies [1, 2].

Among depressed patients, serum concentrations of IL-17 were associated with poorer cognitive status, consistent with neurodegenerative roles of IL-17 in animal cerebral ischemia models [7]. The significant interaction between depression and IL-17 concentrations predicting MMSE scores suggests that depression may confer neural vulnerability to IL-17 mediated inflammatory pathways. Possible bases for this interaction remain speculative; poststroke depression may be associated with central nervous system (CNS) inflammation that could exacerbate IL-17 expression by T cells when they infiltrate the brain and/or with neurotrophic/neuroprotective deficits that might impair neural resilience to IL-17 mediated neurodegenerative pathways [26].

Additional findings from the present study suggest that IL-17 may be related to the depletion of regulatory T cells (Tregs) and to augmented oxidative stress among subjects with depressive symptoms. Some IL-17 secreting cells (Th17 cells) share a common lineage with regulatory T cells and the expansion of Th17 cells may occur at the expense of Tregs [9]. Among subjects with depressive symptoms, higher IL-17 concentrations were associated with lower IL-10 concentrations, suggesting that poststroke depression may be associated with susceptibility to Treg depletion due to a Th17 response. This may exacerbate neurodegenerative damage, to the detriment of cognitive function, since Tregs are thought to be beneficial after stroke due to their secretion of the anti-inflammatory and neuroprotective cytokine IL-10 into the postinfarct brain [9]. Previously, a polymorphism in the promoter region of the IL-10 gene has been associated with poststroke depression [24], which would be consistent with vulnerability to low IL-10 production among depressed patients.

Given the ability of IL-17 to disrupt the blood brain barrier and contribute to neurodegeneration through increased production of reactive oxygen species [11], LPH associated with IL-17 could reflect peroxidation of blood brain barrier or CNS lipids. In a previous study, serum LPH concentrations were associated with subtle damage to cerebral white matter in patient with bipolar disorder but not in nondepressed controls [17]. The basis for the observation of a relationship between concentrations of IL-17 and LPH specifically among subjects with depressive symptoms after stroke requires further investigation. Findings from a recent study suggested that Th17 cells may be more reactive to oxidized lipids in stroke patients, but depressive or cognitive symptomatology was not assessed in that study [8]. The present results might also reflect greater activation of the NLRP3 inflammasome in peripheral blood mononuclear cells from subjects with depression, as suggested in a recent study of depressed and nondepressed medically healthy subjects [27]. NLRP3 inflammasome activity results in maturation and secretion of IL-1*β* or IL-18, and NLRP3 inflammasome assembly can be promoted by reactive oxygen species generated by IL-17. In turn, IL-1*β* or IL-18 resulting from NLRP3 activity can stimulate IL-17 secretion from Th17 or *γ* *δ*T cells, which may result in a feedforward loop that sustains inflammatory cytokine secretion [28–30]. This would also be consistent with the findings of one previous study, in which elevated IL-18 concentrations were associated with poststroke depression [25]. More clinical data will be required in order to replicate these findings and to establish roles of these inflammatory and oxidative stress markers in neurodegenerative pathways and their relationships with depressive and cognitive symptoms.

Although these data suggest adequate power to detect the effects observed for relationships between IL-17, cognitive status, and oxidative stress among patients with depressive symptoms, the results are limited by a small sample size. Potential bias may have been introduced at the level of recruitment although the demographics of the included subjects do not differ substantially from those generally seen at our site. Replication in larger cohorts including larger numbers of patients with elevated IL-17 concentrations will thus be informative. Although protein degradation over the course of storage at −80°C may have affected assayed concentrations, the time between phlebotomy and assay did not affect the main results. The present study measured cytokines at a single time point with variable poststroke sampling times, potentially contributing to heterogeneity in the findings; however, cytokine concentrations were not associated with time since stroke in this sample. Nevertheless, future studies should delineate the time course of IL-17 elevations and the relative significance of elevated IL-17 concentrations specifically in acute, subacute, and chronic stages of stroke. Serum cytokine measurements are limited by variable systemic release and half-lives in circulation, and therefore they may not reflect CNS concentrations; however, peripheral T cells are known to enter the brain after stroke and the present results support previous findings to suggest that a peripheral IL-17 bias may be clinically relevant [10]. While the MMSE is largely used as a screening instrument, it has been validated and used extensively in stroke [5, 6, 15]. The MMSE is sensitive to clinically meaningful cognitive impairment, and it is relatively stable over time after stroke [31]. Finally, while the CES-D has been shown to have excellent concurrent validity with diagnostic criteria for depressive episodes and high accuracy in screening, future studies might confirm the present findings using a structured clinical interview for major depressive disorder criteria.

## 5. Conclusion

These preliminary clinical data would be consistent with vulnerability to IL-17 mediated neurodegenerative pathways in patients with depressive symptoms. The related mechanisms may involve an imbalance between pro- and anti-inflammatory activity and augmented oxidative stress, which may help to characterize poststroke depression and associated cognitive susceptibility.

## Figures and Tables

**Table 1:** Clinical demographic characteristics.

	CES-D < 16 *n* = 28	CES-D ≥ 16 *n* = 19	*X* ^2^ or *t*	*P*
Demographics				
Age (mean ± SD)	68.8 ± 14.1	76.2 ± 14.2	1.77	0.08
Sex (% male)	60.7	68.4	0.29	0.59
Living alone (%)	42.9	31.6	0.61	0.43
Level of education > high school (%)	92.3	94.4	0.08	0.78
History of depression (%)	3.6	18.8	2.17	0.14
Vascular risk factors				
Hypertension (%)	71.4	94.7	3.97	0.05
Diabetes (%)	21.4	36.8	1.34	0.25
Dyslipidemia (%)	60.7	84.2	2.99	0.08
Obesity (BMI ≥ 30) (%)	14.3	21.0	0.37	0.55
Smoking (%)	17.8	21.1	0.08	0.79
Concomitant medications				
Antidepressant use (%)	7.1	15.8	0.89	0.35
ASA use (%)	62.9	73.7	0.58	0.45
NSAID use (other than ASA) %	7.4	21.1	1.83	0.18
Stroke characteristics				
Weeks since stroke (mean ± SD)	3.4 ± 5.3	3.4 ± 4.8	0.04	0.97
NIHSS scores (mean ± SD)	4.6 ± 4.5	5.4 ± 4.7	0.63	0.53
Lesion location				
Anterior (%)	10.7	15.8	0.26	0.61
Posterior (%)	50.0	26.3	2.64	0.10
Intermediate (%)	10.7	26.3	1.95	0.16
Extending (%)	25.0	31.6	0.25	0.62
Lesion side				
Left (%)	46.4	42.1	0.09	0.77
Right (%)	50.0	57.9	0.28	0.60
Bilateral (%)	3.6	0.0	0.69	0.41
Lesion volume (cm^3^) (mean ± SD)∗	28.2 ± 63.0	20.6 ± 47.5	0.65	0.52

*X* ^2^ or *t* values and corresponding  *P*  values reflect results of Pearson's Chi-squared tests for categorical variables and independent *t*-tests for continuous variables.

*n = 21 nondepressed and *n* = 15 depressed.

BMI: body mass index; CES-D: Center for Epidemiological Studies Depression; NIHSS: National Institutes of Health Stroke Scale; NSAID: nonsteroidal anti-inflammatory; ASA: acetylsalicylic acid.

**Table 2:** Serum assay results.

Serum analytes	Median (IQR)		t*	*P*
	CES-D < 16 *n* = 28	CES-D ≥ 16 *n* = 19		
IL-17 (pg/mL)	40.4 (32.8–52.2)	38.0 (33.2–50.7)	0.66	0.52
IL-23 (pg/mL)	300 (273–438)	262 (192–382)	1.81	0.08
IL-10 (pg/mL)	0.20 (0.20–2.41)	0.20 (0.20–2.07)	0.10	0.92
IFN-*γ* (pg/mL)	0.85 (0.18–1.00)	1.00 (0.23–1.00)	0.20	0.84
LPH (nmol/mL)	11.2 (8.25–13.0)	9.06 (7.63–10.5)	0.84	0.41

*t values and corresponding  *P*  values reflect results of Student's *t*-test using log transformed values.

Percentages of analyte concentrations returned below the limit of detectability were 0% for IL-17, IL-23, and LPH, 66% for IL-10, and 30% for IFN-*γ*.

CES-D: Center for Epidemiological Studies Depression; IQR: interquartile range; LPH: lipid hydroperoxides.
